# Tango's maximized excess events test with different weights

**DOI:** 10.1186/1476-072X-4-32

**Published:** 2005-12-15

**Authors:** Changhong Song, Martin Kulldorff

**Affiliations:** 1 Department of Statistics, University of Connecticut, Storrs, CT, 06269, USA; 2 Department of Ambulatory Care and Prevention, Harvard Medical School and Harvard Pilgrim Health Care, 133 Brookline Avenue, 6th Floor, Boston, MA 02215, USA

## Abstract

**Background:**

Tango's maximized excess events test (*MEET*) has been shown to have very good statistical power in detecting global disease clustering. A nice feature of this test is that it considers a range of spatial scale parameters, adjusting for the multiple testing. This means that it has good power to detect a wide range of clustering processes. The test depends on the functional form of a weight function, and it is unknown how sensitive the test is to the choice of this weight function and what function provides optimal power for different clustering processes. In this study, we evaluate the performance of the test for a wide range of weight functions.

**Results:**

The power varies greatly with different choice of weight. Tango's original choice for the weight function works very well. There are also other weight functions that provide good power.

**Conclusion:**

We recommend the use of Tango's MEET to test global disease clustering, either with the original weight or one of the alternate weights that have good power.

## Background

Many tests for spatial randomness that adjust for a heterogeneous background population have been proposed. These test statistics are used to test whether the geographical distribution of disease is random or not. They are also used in many other areas such as geomorphology, ecology, genetics and geography (See, e.g., Fotheringham et al. [1], Gatrell et al. [2], Ruiz-Garcia [3], Aubry and Piegay [4], Clark and Richardson [5], Liebhold and Gurevitch [6], Gustine and Elwinger [7], Meirmans et al. [8]).

Among these test statistics, some are global clustering tests used to evaluate the presence of clustering throughout the study region. Others are used to detect and evaluate local clusters. In this paper, we are only concerned with the former. Examples of global clustering tests are Tango's maximized excess events test (*MEET*) [9], Cuzick and Edwards' *k *nearest neighbors (*k*-NN) [10] and Moran's *I *[11].

When we are using a global clustering test, it is important that it has good statistical power. We have previously [12,13] evaluated the power of seven global clustering tests: Besag-Newell's *R *[14], Bonetti-Pagano's *M *statistic [15], Cuzick-Edwards' *k*-NN, Moran's *I*, Swartz' Entropy test [16], Tango's *MEET *and Whittemore's test [17]. The power varies greatly for different test statistics and Tango's MEET has the best power overall.

Tango's *MEET *depends on a weight function. Tango proposed a distance based exponential weight function for *MEET*', but other choices of weights are also possible. In this paper, we evaluate Tango's *MEET *using different weight functions. Nine weight functions are evaluated, and the power varies greatly with different choice of weight.

## Methods

### Notation

Denote *c*_*i *_as the number of cases in county *i*, *n*_*i *_as the population size of county *i*, *C *as the total number of cases, *N *as the total population size, *H *as the total number of counties, *d*_*ij *_as the distance between county *i *and *j*, *u*_*j*(*i*) _as the population size in county *i *and its *j *nearest neighbors. The maximum distance between county *i *and the other counties under study is denoted by *dmax*_*i *_= *max*_1≤*j*≤*n*_*d*_*ij*_.

### Tango's *MEET*

Tango's [9] MEET is a maximized version of Tango's excess events test (EET) [18]. We first describe the latter.

For a given weight function *w*_*ij*_, Tango's *EET *is a weighted sum of excess events defined as



Tango proposed two distance based exponential weight functions [9] and [18], where λ is a measure of the spatial scale of clustering. To avoid confusion with other weight functions, we denote the *EET *defined by these two distance based exponential weight functions as



and



*DE*1*_EET *and *DE*2_*EET *depends on the scale parameter λ. To be able to detect clustering irrespectively of its geographical scale, Tango [9] proposed the maximized excess events test (*MEET*). We use notation *DE*1*_MEET *and *DE*2*_MEET *to denote the maximized tests of *DE*1_*EET *and *DE*2_*EET *respectively, which are defined as



and



where *de*1_*eet*(λ) and *de*2_*eet*(λ) are the observed values of *DE*1_*EET*(λ) and *DE*2_*EET*(λ) conditioning on λ. U is an upper limit on λ. Basically, the maximized test is using the minimum of the profile p-values as the test statistics adjusting for the multiple testing resulting from the many parameter values considered.

### Alternative weight functions

Since Tango's *MEET *performs very well, it is of interest to evaluate other potential weight functions, of which there are many. Nine weight functions including Tango's distance based exponential weights are evaluated in this paper. Five of them depend on a spatial scale parameter while four of them do not.

For all weights function, the weight decreases with increasing distance. The metric used for the decrease is different though. For example, the weight may be defined on Euclidean distance and depends only on distance. It may also be adjusted with population density, so that the weight declines faster in urban than in rural areas. We can also define the weight in terms of spatial contiguity of counties irrespective of the population density. Other choices of weight functions may take geographical or population size into consideration. We describe our weight functions next.

#### Population density adjusted exponential weight

The scale of the spatial clustering usually depends on the underlying population. It will be reasonable to adjust the weight function with the underlying population density. We define this weight function as , where  and *m*_*i *_= *max*{*j *: *u*_*j*(*i*) _≤ *k*}. The parameter *k *is set by the user and can be viewed as a population measure for the clustering. Note that for a given *k*, λ_*i *_in the rural area, which has a small population density, is larger. This means the hazard rate in the rural area decreases slower than that of urban area as the Euclidean distance becomes large. Usually, large *k *is more sensitive to large clusters and small *k *is more sensitive to small clusters. To study the strength of the parameter, we take the value of *k *equal to 50%, 25%, 10% and 5% of the overall population. We denote the test statistic with this weight function as *PE_EET *and



#### Nearest neighbor adjusted weight

Another potential weight function, based on the nearest neighbors property, is defined by , where *l *indicates that county *j *is the *l*th closest county to county *i*. So the weight for county *i *itself is 1, the weight for its closest neighbor is , the weight for its second closest neighbor is , and so on. This weight function is based on spatial contiguity of counties adjusted with distance. It may be desirable when the hazard risk does not decrease proportionally with distance. A small value of the parameter *s *will give more weight to the counties far from county *i*, while a large *s *will give more weight to county *i *and its closest neighbors. We set *s *= 0.1, 0.5, 1, 2, 8 to study the property of the parameter. We denote the *EET *test statistic with this weight as



#### Distance adjusted weight

The next weight function is defined by . This weight function gives more weight to the counties that are geographically close to each other. The 1 in the denominator is used to adjust the weight so that when the distance is very small, the weight will not be too large. *H *is used to adjust the weight with the total number of counties. The *EET *test with this weight is written as



#### Distance and area adjusted weight

For a different spatial statistical method, Gangnon and Clayton [19] used the weight function , where *a*_*i *_denotes the area of county *i*, *A *denotes the total area of all counties. The test statistic is denoted as



#### Distance and population adjusted weight

By replacing the area size in the above weight function with population size, we get another weight function . The test statistic is



#### Adjacent neighbor weight

If we define two individual persons to be neighbors if they are in the same county or neighboring county, then we can get the stepwise weight function



Test statistic with this weight is



#### Population based weight

Another possible weight function is to use the product of the proportion of the corresponding counties to the total population, so that . The test statistic is then



Note that this population based weight does not take into account any distance information between counties.

#### Maximized tests over spatial scale parameters

Three *EET *tests, *PE_EET*, *NN_EET *and *D_EET*, depend on a parameter. By using Tango's maximization technique, which uses the minimum profile p-value of *EET *for the parameter, we get the maximized tests for these three tests. For *PE_EET*(*k*), the *MEET *is defined as



where *pe_eet*(*k*) is the observed value of the excess events test statistic conditioning on *k*, and *V *is an upper limit on *k*. *H*_0 _denotes the null hypothesis of no spatial correlation for the data. Our implementation of the test is carried out by choosing *k *as 5%, 10%, 15%, ..., 50% of the population.

Similarly, for *NN_EET*(*s*), we define the *MEET *as



where *nn_eet*(*s*) is the observed value of the excess events test statistic conditioning on *s*. The implementation of this test is carried out by choosing *s *as 0.1, 0.25, 0.5, 1, 1.5, 2, 4, 8, 10. The *MEET *for *D_EET*(*s*) is similar to *NN_EET*(*s*), and it is defined as



The implementation of this test is carried out by the same collection of *s *as for *NN_MEET*.

### Benchmark data

To evaluate statistical power, we used a collection of benchmark data sets based on the 1990 female population in the 245 counties and county equivalents in the northeastern United States, consisting of the states of Maine, New Hampshire, Vermont, Massachusetts, Rhode Island, Connecticut, New York, New Jersey, Pennsylvania, Delaware, Maryland and the District of Columbia. The benchmark data has been described in detail elsewhere [13]. It can be downloaded at ''.

Under the null hypothesis of no clustering, 99,999 random data sets were generated by randomly allocating 600 cases to various counties, with the probabilities proportional to the county population. The null data is used to estimate the critical values, which is the cut-off point for the significance.

**Figure 1:**
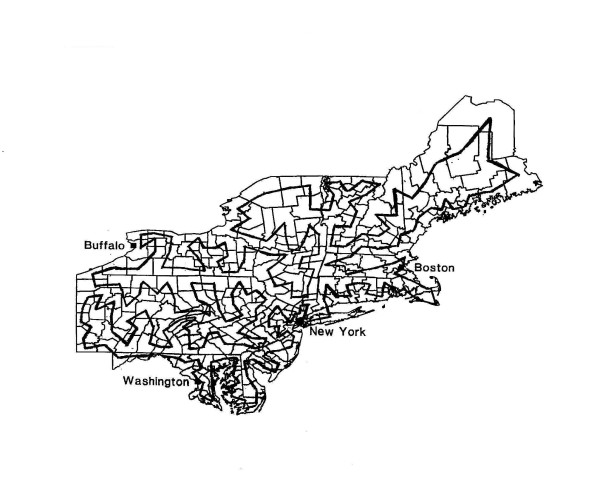
Hamiltonian chain of counties used for the global chain clustering.

For each clustering model, 10,000 random data sets were used to estimate the power. The counties are tied together sequentially on a chain that passes through each county exactly once, after which it reconnects with the first county on the chain, forming a Hamiltonian cycle. A map of the Hamiltonian cycle used has been illustrated in figure 1. The clusters are generated by first locating 300 cases randomly on the map under the null hypothesis. Then each of these original cases generates one new case for a total of 600. There are three types of clustering models with the distance between the twins along the chain being either constant or exponentially distributed with different means. For the first type of clustering models, the distance between twins is zero, which means the twins are always in the same county. For the second type of clustering, six clustering models were constructed by setting the distance between twins to be fixed with the mean corresponding to 0.5%, 1%, 2%, 4%, 8% and 16% of the overall population along the chain. For the third type of clustering models, the distance was set to be exponentially distributed and span over 0.5%, 1%, 2%, 4%, 8% and 16% of the overall population size. The Hamiltonian cycle does not imply that the disease itself spreads around the chain, just that twin cases are located in either of the two directions, as defined by the chain.

## Results

Table 1 shows the estimated power of Tango's *EET *and *MEET *with different choice of weight. The highest power for each clustering model is highlighted. The power varies greatly with different choice of weight. *PE_MEET*, *DE*1_*MEET*, *DE*2*_MEET*, *NN_MEET *and *D_MEET *all have very good power. *DA_EET*, *DP_EET *and *N_EET *have good power for some clustering models, but not as good as the *MEET *tests. *P_EET *does not perform well. All tests have lower power as the distance between twins increases since there is less clustering in the data.

**Table 1:** Power of the test statistics for the global twin clustering. The row variable denotes the test statistics. The column variable denotes the clustering models. The last column is the average power for each test statistic.

	Fixed distance							Exponential distance						
	0.00	0.5%	1%	2%	4%	8%	16%	0.5%	1%	2%	4%	8%	16%	average
*P_EET*	0.16	0.15	0.14	0.12	0.10	0.07	0.04	0.15	0.14	0.13	0.11	0.09	0.07	0.11
*DA_EET*	0.91	0.35	0.19	0.09	0.06	0.05	0.05	0.48	0.32	0.20	0.13	0.08	0.06	0.23
*DP_EET*	0.64	0.42	0.27	0.12	0.07	0.06	0.04	0.46	0.34	0.23	0.15	0.10	0.07	0.23
*N_EET*	0.71	0.57	0.46	0.30	0.13	0.07	0.05	0.61	0.51	0.38	0.25	0.15	0.09	0.33
														
*NN_MEET*	**0.99**	0.68	0.45	0.27	0.16	0.10	**0.07**	0.79	0.62	0.42	0.27	0.17	0.10	0.39
*D_MEET*	**0.99**	0.67	0.42	0.24	0.15	0.10	**0.07**	0.78	0.61	0.41	0.26	0.16	0.10	0.38
*PE_MEET*	0.98	**0.73**	**0.51**	**0.31**	0.17	0.10	0.06	**0.81**	**0.65**	**0.46**	**0.29**	**0.18**	**0.11**	**0.41**
*DE*1_*MEET*	**0.99**	0.64	0.41	0.26	**0.18**	**0.12**	**0.07**	0.75	0.57	0.39	0.26	0.17	**0.11**	0.38
*DE*2_*MEET*	**0.99**	0.62	0.41	0.26	0.17	0.11	0.06	0.74	0.56	0.38	0.25	0.17	**0.11**	0.37

In Table 2, we present the estimated power of the four weight functions that depend on a parameter. For each weight function, we choose different parameter values that can represent its overall strength and compare them with the maximized test. For *DE*1*_EET*(λ), *DE*2*_EET*(λ) and *PE_EET*(*k*), large parameter value is more sensitive to clustering with large scale, while small parameter value is more sensitive to clustering with small scale. For *NN_EET*(*s*) and *D_EET*(*s*), large parameter value is more sensitive to clustering with small distance, while small parameter value is more sensitive to clustering with large distance. *DE*1_*EET*(λ) and *DE*2_*EET*(λ) have very similar performance, but *DE*2_*EET*(λ) is more sensitive to the choice of parameter. For all the five tests, the maximized tests have less power compared to the maximum power that can be obtained by the *EET *test with an appropriately chosen parameter, but the maximized test has reasonably good power overall. For example, for the fixed 1% distance clustering, the maximized test *PE_MEET *has the power of 0.73. The original test *PE_EET*(*k*) has the power of 0.75 with *k *equal to 5% or 10% of the population, but it has a low power of 0.42 when *k *is 50% of the population.

**Table 2:** Power of the test statistics for the global twin clustering using different spatial scale parameters. The row variable denotes the test statistics. The column variable denotes the clustering models. The last column is the average power for each test statistic.

		Fixed distance						Exponential distance						
	0.00	0.5%	1%	2%	4%	8%	16%	0.5%	1%	2%	4%	8%	16%	average
*DE*1_*EET*(λ) with														
λ = 66, 000	0.37	0.32	0.29	0.26	0.20	**0.14**	**0.08**	0.32	0.30	0.26	0.22	0.17	0.11	0.23
λ = 32, 000	0.44	0.36	0.32	0.27	0.21	**0.14**	**0.08**	0.37	0.33	0.29	0.23	0.17	**0.12**	0.26
λ = 15, 000	0.58	0.43	0.37	**0.29**	**0.22**	**0.14**	0.07	0.46	0.40	0.33	0.25	**0.18**	**0.12**	0.29
λ = 4, 000	0.95	0.59	**0.41**	0.26	0.16	0.10	0.06	0.68	0.54	0.38	0.25	0.16	0.10	0.36
λ = 500	**1.00**	**0.67**	0.34	0.13	0.06	0.06	0.06	**0.80**	**0.60**	0.37	0.20	0.12	0.07	0.34
*DE*1_*MEET*	0.99	0.64	**0.41**	0.26	0.18	0.12	0.07	0.75	0.57	**0.39**	**0.26**	0.17	0.11	**0.38**

*DE*2_*EET*(λ) with														
λ = 66, 000	0.30	0.28	0.27	0.25	0.20	**0.14**	**0.08**	0.28	0.27	0.25	0.21	0.16	**0.11**	0.22
λ = 32, 000	0.42	0.37	0.33	**0.28**	**0.21**	0.13	0.06	0.38	0.34	0.29	0.23	**0.17**	**0.11**	0.25
λ = 15,000	0.68	0.47	0.38	**0.28**	0.18	0.11	0.06	0.51	0.43	0.33	0.24	0.16	0.10	0.30
λ = 4, 000	0.99	0.61	0.34	0.17	0.10	0.07	0.05	0.74	0.55	0.35	0.21	0.13	0.08	0.34
λ = 500	**1.00**	**0.66**	0.33	0.13	0.06	0.06	0.06	**0.80**	**0.59**	0.36	0.19	0.11	0.07	0.34
*DE*2_*MEET*	0.99	0.62	**0.41**	0.26	0.17	0.11	0.06	0.74	0.56	**0.38**	**0.25**	**0.17**	**0.11**	**0.37**

*PE_EET*(*k*) with														
*k *= 50%*population*	0.48	0.42	0.37	0.31	**0.22**	**0.13**	**0.07**	0.43	0.38	0.33	0.25	**0.18**	**0.11**	0.28
*k *= 25%*population*	0.72	0.57	0.47	**0.33**	0.20	0.10	0.05	0.60	0.52	0.41	0.28	**0.18**	**0.11**	0.35
*k *= 10%*population*	0.96	**0.75**	**0.53**	0.27	0.11	0.06	0.05	0.81	0.66	**0.47**	0.28	0.15	0.09	0.40
*k *= 5%*population*	**0.99**	**0.75**	0.45	0.17	0.06	0.06	0.06	**0.84**	**0.67**	0.43	0.23	0.13	0.07	0.38
*PE_MEET*	0.98	0.73	0.51	0.31	0.17	0.10	0.06	0.81	0.65	0.46	**0.29**	**0.18**	**0.11**	**0.41**

*NN_EET*(*s*) with														
*s *= 0.1	0.72	0.52	0.42	**0.32**	**0.22**	**0.13**	**0.07**	0.56	0.48	0.38	0.28	**0.19**	**0.12**	0.34
*s *= 0.5	0.93	0.64	**0.47**	0.31	0.18	0.11	0.06	0.71	0.59	0.43	**0.29**	0.18	0.11	**0.39**
*s *= 1	0.99	**0.70**	0.46	0.25	0.13	0.08	0.06	0.79	**0.64**	**0.44**	0.27	0.16	0.09	**0.39**
*s *= 2	**1.00**	**0.70**	0.40	0.17	0.08	0.06	0.06	**0.82**	0.63	0.41	0.23	0.13	0.08	0.36
*s *= 8	**1.00**	0.66	0.33	0.13	0.06	0.06	0.06	0.80	0.59	0.36	0.19	0.11	0.07	0.34
*NN_MEET*	0.99	0.68	0.45	0.27	0.16	0.10	**0.07**	0.79	0.62	0.42	0.27	0.17	0.10	**0.39**

*D_EET*(*s*) with														
*s *= 0.1	0.91	0.57	0.41	**0.28**	**0.19**	**0.13**	**0.07**	0.65	0.52	0.39	0.27	**0.19**	**0.11**	0.36
*s *= 0.5	0.99	0.68	**0.44**	0.25	0.15	0.10	0.06	0.78	**0.62**	**0.43**	**0.28**	0.17	0.10	0.37
*s *= 1	**1.00**	**0.69**	0.39	0.18	0.09	0.07	0.06	**0.81**	**0.62**	0.41	0.23	0.14	0.08	**0.38**
*s *= 2	**1.00**	0.67	0.34	0.13	0.06	0.06	0.06	0.80	0.60	0.37	0.20	0.12	0.07	0.34
*s *= 8	**1.00**	0.66	0.33	0.13	0.06	0.06	0.06	0.80	0.59	0.36	0.19	0.11	0.07	0.34
*D_MEET*	0.99	0.67	0.42	0.24	0.15	0.10	**0.07**	0.78	0.61	0.41	0.26	0.16	0.10	**0.38**

## Discussion

In this paper, we evaluated Tango's *EET *and *MEET *using both used and unused weight functions. The power can vary greatly with the choice of weight. This indicates that for global clustering test, consideration of weight is important. For the weight functions that incorporate good distance information, the power of the test is much better than the weight functions that do not incorporate the spatial relationship between counties.

With reasonable parametric distance based weights, the power of Tango's *MEET *is rather robust. For this study, *PE_MEET*, *DE*1_*MEET*, *DE*2_*MEET*, *NN_MEET *and *D_MEET *all have good power, and their average power for all clustering models considered are very similar.

Tango's *DE*1_*MEET *and *DE*2*_MEET *scan over the study area by distance. These two tests have similar performance. Both tests are based on the summation of the weighted excess events and they collect clustering information throughout the map, which makes them good global tests. Previous studies [12,13] indicated that *DE*2_*MEET *perform well when the cluster is large in population size.

For the clustering models considered in this paper, *PE_MEET *performs a little better than the tests with other weights. The reason for this may be due to the way that the data were generated based on the population density. We believe some of the test statistics may have better strength under other alternate models. We use the female population in the 245 counties and county equivalent in Northeastern United States as the underlying population. It is possible that the relative strength of the various test statistics may be different for other underlying population or different alternative clustering models. The type of power evaluations done in this paper are, in spite of these limitations, very important. For practical applications, the power estimates presented in this paper provides some help when we choose a test.

## Conclusion

The power of Tango's *MEET *varies greatly with different choice of weight. In general, with reasonable parametric distance based weights, the power of Tango's *MEET *is robust. Tango's original choice for the weight function works well. At the same time, there are also other weight functions for which the test has good power.

## List of abbreviations

EET: Excess Events Test.

MEET: Maximized Excess Events Test.

## Authors' contributions

CH and MK jointly designed the study and chose the methods for evaluation. CH programmed the S-Plus code, carried out the power calculations and wrote the first draft of the manuscript. Both authors interpreted the results and wrote the final version of the paper.
